# Inflammatory Mediators of Leprosy Reactional Episodes and Dental Infections: A Systematic Review

**DOI:** 10.1155/2015/548540

**Published:** 2015-08-03

**Authors:** D. C. B. Cortela, A. L. de Souza Junior, M. C. L. Virmond, E. Ignotti

**Affiliations:** ^1^School of Health Sciences, State University of Mato Grosso, São João Street, S/N, 78000-000 Cáceres, MT, Brazil; ^2^Research Department, Instituto Lauro de Souza Lima, Comandante J. R. Barros Rodovia Km 225/226, 17034-971 Bauru, SP, Brazil

## Abstract

Reactional episodes in leprosy are a result of complex interactions between the immune system, *Mycobacterium leprae*, and predisposing factors, including dental infections. To determine the main inflammatory mediators in the immunopathological process of dental infections and leprosy reactions, we conducted a systematic review of primary literature published between 1996 and 2013. A three-stage literature search was performed (Stage I, “leprosy reactions” and “inflammatory mediators”; Stage II, “dental infections” and “inflammatory mediators”; and Stage III, “leprosy reactions,” “dental infections,” and “inflammatory mediators”). Of the 911 eligible publications, 10 were selected in Stage I, 68 in Stage II, and 1 in Stage III. Of the 27 studied inflammatory mediators, the main proinflammatory mediators were IL-6, IFN-*γ*, TNF-*α*, IL-1*β*, and IL-17; the main anti-inflammatory mediators were IL-10 and IL-4. Serum IL-6 and TNF-*α* concentrations were significant during periodontal and reactional lesion evolution; IFN-*γ* and IL-1*β* were associated with types 1 and 2 reactions and chronic periodontal disease. The proinflammatory mediators in dental infections and leprosy reactions, especially IL-6 and TNF-*α*, were similar across studies, regardless of the laboratory technique and sample type. IFN-*γ* and IL-1*β* were significant for leprosy reactions and periodontal diseases. This pattern was maintained in serum.

## 1. Introduction

Leprosy reactions are sudden acute immune-inflammation episodes against* Mycobacterium leprae* superimposed on the chronic course of leprosy. They predominate in individuals classified as multibacillary and are responsible for irreversible nerve damage, increasing the disease burden and associated stigma [1].

Identified as type 1 reactions (T1Rs), type 2 reactions (T2Rs), or neurological reactions, leprosy reactions show distinct immunological characteristics and may occur before or during treatment as well as up to 5 years or more after the conclusion of polychemotherapy. A T1R is clinically characterized by the increase and exacerbation of preexisting lesions with no involvement of the individual's general condition. In a T2R, nerve involvement is less frequent; the individual presents with general malaise, fever, and systemic involvement, which is not restricted only to the skin. Isolated neuritis results in symptoms and neurological signs without the cutaneous manifestations of T1Rs and T2Rs; in the absence of pain, they are called silent neuritis [2–4]. Approximately 25–50% of sick individuals can develop reactions [5–8].

Among individuals with borderline leprosy, 30% show a risk of T1R; the incidence is significantly higher in borderline-borderline and borderline-lepromatous (BL) cases than in borderline tuberculoid cases. In contrast, T2R occurs more frequently in individuals with lepromatous leprosy (LL), affecting 20% of LL cases and 10% of BL cases [9, 10].

Studies published in the past 5 years have addressed the possible relationship between the occurrence of reactional episodes and dental infections [11–14]. The oral health conditions in individuals with leprosy are poor, that is, high rates of caries and periodontal disease (PD) [15–20], with little involvement of dentists to control these diseases [11, 21].

Leprosy reactions and dental infections have some common characteristics. They are both slowly evolving chronic infections, modulated by a number of inflammatory and immunopathological events resulting from the interaction between bacteria and their products and the host immune response. Both the complications of leprosy and extent and severity of PD manifest as secondary damage, arising from an unsuccessful defense mechanism of the host [22–24]. Common and important mediators expressed in both conditions include IL-1, IL-1*β*, IL-4, IL-6, IL-8, IL-10, TNF-*α*, and IFN-*γ* [25–29].

The release of cytokines in response to oral bacteria is among the mechanisms underlying the systemic effects of periodontitis [30, 31]. Motta et al. [12–14] investigated the role of dental infections in the triggering, maintenance, or exacerbation of reactive episodes and emphasized the possible role of IL-6, IL-10, and IL-1 in these events. However, there is need for additional studies to understand this possible interaction.

The hypothesis of a close relationship between oral diseases and certain systemic conditions is not new. The scientific evidence in dentistry and medicine has corroborated the bidirectional relationship between an individual's general health and oral health as well as specific oral diseases, such as PDs [32–34].

Considering the scarcity of studies aimed at investigating the relationship between dental infections and leprosy reactions and the possibility of a systemic effect of cytokines in the immunopathological mechanisms of these diseases, this systematic review aimed at analyzing scientific publications reporting the inflammatory mediators involved in the immunopathological processes of dental infections and leprosy reactions.

## 2. Materials and Methods

### 2.1. Type of Study

This was an exploratory systematic review of the primary literature on inflammatory mediators involved in the immunopathological process of reactional episodes in leprosy and dental infections.

### 2.2. Data Sources and Time Period

A search of the literature was conducted between January and December 2013 in the following electronic databases: (i) national database (BBO Dental/Brazil, Spanish Bibliographic Index of Health Sciences/IBECS, and Scientific Electronic Library Online/SciELO); (ii) international database (Latin American and Caribbean Health Sciences/LILACS, US National Library of Medicine/PubMed, and U.S. National Library of Medicine's bibliographic database/MedLine); and (iii) the cochrane library.

### 2.3. Search Strategy and Selection of Articles

Considering that the term “periodontal medicine” was first used in dentistry in 1996 to designate the branch of periodontology addressed to the investigations of the bidirectional relationships between PDs and the general condition of the individual [33, 34], we limited the literature search to studies published between January 1, 1996, and December 31, 2013.

The search strategy was constructed with descriptors in English, Spanish, and Portuguese and considering their synonyms, according to the specificities of the databases.

We identified the descriptors in the health sciences by consulting the DeCS according to Keywords in Context and the Medical Subject Headings (MeSH).

The following MeSH terms and keywords were used:

1. Pulpitis.
2. Gingival diseases, gingivitis.
3. Periodontitis; periodontal diseases, hierarchical term in MeSH: aggressive periodontitis; chronic periodontitis; periapical periodontitis; periodontal abscess; periodontal pocket.
4. Cytokines; interleukin(s) (blood/skin).
5. Inflammation mediators; biological markers; biomarkers.
6. Type I reversal reaction; reversal reaction; erythema nodosum leprosum.
7. Leprosy reaction; leprosy reactions; leprosy reactional.
8. Biopsy.
9. Skin.

The search was conducted in three stages. During Stage I, the bibliographic search was conducted for the terms “*leprosy reactions*” and “*cytokines.*” In Stage II, we used “*dental infections*” and “*cytokines,*” and, in Stage III, we used “*leprosy reactions,*” “*dental infections*,” and “*cytokines*.”

Studies were included that investigated the participation and involvement of cytokines in the inflammatory process of dental infections and/or during the occurrence of leprosy reactions.

We excluded all articles that had any of the following groups: pregnant women, syndromic individuals, smokers, experimental animals, or individuals with systemic diseases or conditions (diabetes, menopause, cardiovascular disease, and chronic renal failure). We also excluded studies involving cell cultures, influence of drugs, the production of cytokines and periodontopathic bacteria, genetic polymorphisms, mutations, case reports, systematic reviews, literature reviews, and meta-analyses.

After the article selection, we constructed a form with the following pieces of information: author and year, study population (sample size, age, type of dental infection, and/or reactional episode), laboratory techniques used in the studies, cytokines analyzed, and additional relevant results.

Exploratory analyses using tables, figures, and flowcharts were conducted.

## 3. Results

**Figure 1:**
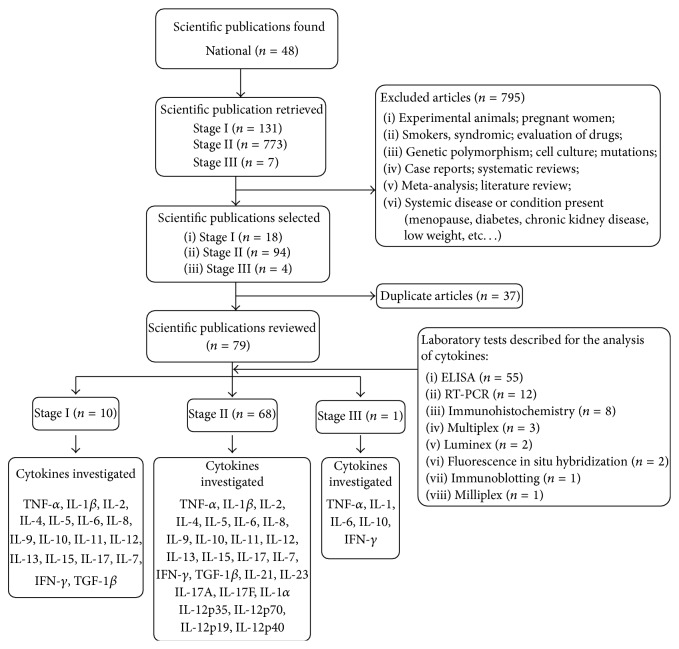
Flowchart of the selection of scientific articles published between January 1, 1996, and December 31, 2013, regarding inflammatory mediators involved in leprosy reactional episodes and dental infections. Stage I (bibliographic search for inflammatory mediators/leprosy reactions); Stage II (bibliographic search for inflammatory mediators/dental infections); Stage III (bibliographic search for inflammatory mediators/dental infections/leprosy reactions).

We identified 911 publications dated between January 1, 1996, and December 21, 2013, of which we excluded 795; a further 37 articles were duplicates. We selected the remaining 79 publications for analysis: 10 articles (12.7%) in Stage I, 68 articles (86.0%) in Stage II, and 1 article (1.3%) in Stage III. In these articles of dental infections and the occurrence of reactional episodes, the 27 researched inflammatory mediators, independent of the laboratory technique and type of sample, were TNF-*α*, IFN-*γ*, IL-1/IL-1*β*, IL-1*α*, IL-2, IL-4, IL-5, IL-6, IL-7, IL-8, IL-9, IL-10, IL-11, IL-12, IL-13, IL-12p35, IL-12p70, IL-12p40, IL-15, IL-17, IL-17A, IL-17F, IL-18, IL-21, IL-23, IL-23 p19, and TGF-1*β* (Figure 1).

The use of ELISA (69%) and RT-PCR (15%) to detect the inflammatory mediators was more frequently reported (Figure 1).

Among the studies on dental infection, the most common proinflammatory mediators were IL-1*β* (29 articles), TNF-*α* (25 articles), IL-6 (24 articles), and IFN-*γ* (17 articles), and the most common anti-inflammatory mediator was IL-4 (15 articles). For leprosy reactions, the most common proinflammatory mediators were TNF-*α* (7 articles), IFN-*γ* (5 articles), IL-6 (4 articles), and IL-17 (3 articles), and the most common anti-inflammatory mediators were IL-4 (4 articles) and IL-10 (4 articles) (Table 1).

Of the publications regarding the role of inflammatory mediators in the immunopathological process of dental infections, 10% were associated with periapical lesions (e.g., cyst, periapical granuloma, keratocyst, chronic periapical lesion, radicular cyst, and periapical lesion), 10% were associated with the presence of severe caries and/or pulpitis, and 79% referred to PD.

Of the 19 studies that analyzed the participation of inflammatory mediators in PD, defined as mild, moderate, or severe, or whose definition parameters included only measurements of the periodontal pocket depth, clinical attachment loss, and presence of gingival bleeding, inflammatory mediators were correlated with the clinical parameters of PD. The following cytokines were included in these articles: TNF-*α*, IL-1*β*, IL-2, IL-4, IL-6, IL-8, IL-10, IL-11, IL-13, IL-17, IL-18, IL-23, IFN-*γ*, and IL-12p35 (Table 2).

Laboratory analyses of inflammatory mediators were conducted in serum (11 articles), gingival, pulp, or periapical tissue biopsy (28 articles), and gingival crevicular fluid (GCF) or saliva (29 articles). Only one article presented the analysis of inflammatory mediators in plasma (Table 2).

Only one publication investigated the role of mediators during reactional episodes in individuals with dental infections. We identified higher serum IL-1, IL-6, and IL-10 levels in individuals with leprosy and dental infection compared with individuals with leprosy without dental infection (Table 3).

**Figure 2:**
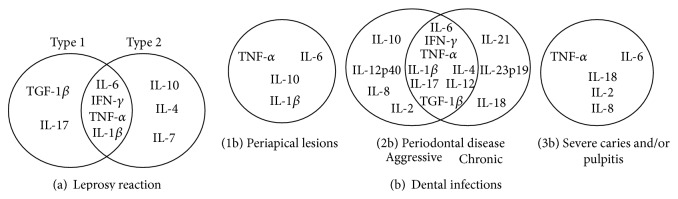
Main inflammatory meditators identified for leprosy reaction (a) and dental infections (b) in articles published between January 1, 1996, and December 1, 2013, and selected for the systematic review.

Among the articles of leprosy reactions, regardless of the type of sample, IFN-*γ*, TNF-*α*, IL-6, and IL-1*β* were involved in T1Rs and T2Rs. In addition, IL-17 and TGF-*β*1 were involved in T1Rs, and the anti-inflammatory mediators IL-10, IL-4, and IL-7 were involved in T2Rs (Figure 2(a)).

IL-6 and TNF-*α* were involved in the immunopathological process of dental infections (Figure 2(b)), which included periapical lesions (Figure 2(b)(1b)), PD (Figure 2(b)(2b)), and severe caries and/or pulpitis (Figure 2(b)(3b)). In studies of aggressive periodontitis (PDag) and chronic PD (CPD), 6 proinflammatory mediators (IL-1*β*, IL-6, IFN-*γ*, TNF-*α*, IL-17, and IL-12) and 2 common anti-inflammatory mediators (TGF-*β* and IL-4) were involved.

**Figure 3:**
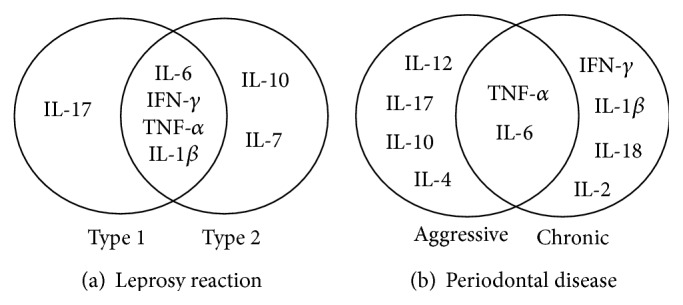
Main serum inflammatory mediators that were identified in leprosy reactions and periodontal diseases, by type, in the articles published between January 1, 1996, and December 31, 2013, that were selected for the systematic review.

Among the common proinflammatory mediators identified in serum during the occurrence of leprosy reactions (a) and PD (b), IL-6 and TNF-*α* were predominant (Figure 3).

In the immune process of T2Rs, only the anti-inflammatory mediators IL-10 and IL-7 were present in serum. During the occurrence of T1Rs and T2Rs, the proinflammatory mediators IL-6, IFN-*γ*, TNF-*α*, and IL-1*β* were detected. IL-17 participated during the occurrence of T1Rs (Figure 3).

In CPD, we also identified IL-*β*1, IFN-*γ*, IL-18, and IL-2 as the proinflammatory mediators in serum. The anti-inflammatory mediators IL-4 and IL-10 were only identified in serum for PDag. For T2Rs and CPD, the common serum proinflammatory mediators included IL-6, IFN-*γ*, TNF-*α*, and IL-*β*1 (Figure 3).

## 4. Discussion

In this systematic review, we identified important pro- and anti-inflammatory mediators involved in the occurrence of dental infections and leprosy reactions, including IL-6, IFN-*γ*, TNF-*α*, IL-1*β*, IL-17, IL-10, and IL-4, which were independent of the laboratory technique and sample. In serum, significant concentrations of IL-6 and TNF-*α* were present during the evolution of periodontitis and reactional lesions, while IFN-*γ* and IL-1*β* were related with T1R, T2R, and CPD.

Such inflammatory mediators are produced by a wide variety of cells during the acute and chronic phases of inflammation, and they have important modulatory and regulatory functions in the inflammatory responses of the immune system. They function together to create a complex network with redundant, synergistic, or antagonistic properties. Furthermore, some molecules are pleiotropic and may have endocrine activity, such as IL-6, TNF-*α*, and IL-1*β* [35, 36].

### 4.1. IL-6

IL-6 is mainly synthesized in the presence of IL-1, TNF-*α*, and lipopolysaccharides that are present in the cell walls of gram-negative bacteria, including the periodontopathogens. It is multifunctional and is present in both the innate and adaptive immune responses, with a key role in the acute immune inflammatory response. It stimulates the T lymphocytes, contributes to the increase of B lymphocytes, and contributes to the production of antibodies in the Th2-cell-mediated immune response [10, 26, 37, 38].

IL-6 and TNF-*α* have been found in biopsy specimens of all individuals with T1R or T2R [39], who have also demonstrated increased levels of IL-6 in serum [26, 40]. Considering its proinflammatory potential and ability to stimulate the production of antibodies, some authors have suggested this cytokine as a valuable prognostic marker for leprosy reactions [7, 9, 36, 37, 40, 41].

In more recent studies, an association between increased plasma IL-6 levels and the occurrence of T1R and T2R has been reported. In T1R, this condition can be explained by the probable participation of cells related to the T1 type response, resulting from nongenetic and/or genetic determinants. In contrast, in T2R, the main determinant for the significant increase in IL-6 seems to be the presence of polymorphisms in the encoding gene of this cytokine [7, 41].

In the present review, regardless of the type of sample used, IL-6 in dental infections was associated with the presence of severe caries, symptomatic periapical lesions, and PD status. As osteoclast-activating factors, IL-6, TNF-*α*, and IL-1*β* are involved in bone resorption during the evolution of PDs [42–50]. IL-6 was correlated with the probing depth and sulcus impairment; it was identified in biopsy specimens, saliva, and gingival fluid, in addition to serum and plasma [42, 43, 46, 47, 49–52]. Individuals with CPD in advanced stages, severe periodontitis, or PDag had significant IL-6 levels [42, 49–51]. The presence of polymorphic variants in the IL-6 gene has indicated an association with the pathogenesis of CPD [53], as well as an increased risk for PDag [54]. Interestingly, this polymorphism seems to have a similar location as that of SNP rs1800795, which is associated with T2R [41].

According to Motta et al. [12], IL-6 is among the mediators possibly involved in the maintenance of reactional episodes, in addition to serum IL-1 and IL-10. Multibacillary individuals with oral infections showed a greater risk for reactions, especially of the erythema nodosum leprosum type, with a clinical improvement of reactional episodes after dental therapy [12, 13, 55]. Recently, it was also observed that dental infections in individuals with leprosy could increase the proinflammatory response mediated by IFN-*γ*, while the opposite effect occurred for the immunoregulatory activity of IL-4, resulting in exacerbation of the inflammatory reaction [14].

### 4.2. IFN-*γ*

Serum IFN-*γ* levels during the occurrence of T1R, T2R, and CPD favor the phagocytic activity in inflammation and amplify the response activity of T cells. It has also been observed in gingival tissue biopsies from lesions of patients with leprosy, saliva, and GCF [43, 45, 49, 50, 56–60]; it is secreted by CD4+ T cells, CD8+ lymphocytes, peripheral blood mononuclear cells, and natural killer (NK) cells, which are also related to periodontal bone loss [14, 26, 61, 62].

High serum IFN-*γ* levels during the reactivation or in excessive acute immune inflammatory responses during the occurrence of reactional episodes have been discussed in the literature; however, its immunoregulatory mechanisms remain unclear. Verhagen et al. [63], when assessing the change of T-cell subsets in the occurrence of T1R and the profile of secreted cytokines, observed a significant amount of Th0 cells with production of both IFN-*γ* and IL-4. However, individuals with T1R recurrence also showed a bias for Th1 with production of IFN-*γ*. In both T1R and, mainly, T2R, there is evidence of the involvement of IFN-*γ* in cellular processes [10, 26, 62, 64–66].

The balance between Th1 and Th2 cells and the change in the serum mediator and skin expression profiles (i.e., IFN-*γ*, TNF-*α*, IL-1*β*, IL-6, and IL-4), in the occurrence of both T1R and T2R, seem to be closely related with the clinical spectrum of the disease. Studies show that, in borderline tuberculoid individuals with T1R, the infiltration of LT CD4+ observed in skin and nerve lesions favored by the synergism between IFN-*γ* and TNF-*α* may possibly contribute to the exacerbated cell-mediated response, resulting in the elimination of mycobacterial antigens and the development of tissue damage. On the other hand, the immunosuppressive activity of IL-4 and IL-10, the increase in IFN-*γ* and TNF-*α*, and also the increase in IL-*β*, and IL-6 observed in BL and LL individuals support the evidence of a systemic inflammatory response in the evolution of T2R [26, 61, 62, 64, 65, 67].

With regard to PDs, there is no consensus on the immunological patterns involved in its pathophysiology. In early/stable periodontal lesions (gingivitis), migration of neutrophils of the junctional epithelium to the gingival sulcus and activation of macrophages and T cells are observed, with a predominance of TNF-*α*, IL-12, and IFN-*γ*, suggesting a cellular response against the pathogens with a Th1 profile and infection control. In advanced/progressive lesions (periodontitis), there are similar proportions of cells with a Th1 profile expressing IFN-*γ* and IL-2 and cells with a Th2 profile expressing IL-4 and IL-6 as reported by Berglundh et al. [68], with combined functioning of these cells in chronic periodontitis. The recruitment of B lymphocytes and the production of immunoglobulins strengthen the Th2 profile signaling [22, 36, 60].

The IFN-*γ* produced by Th1 cells in the initial lesion may contribute to infection control by increasing the phagocytic activity of neutrophils and macrophages. When the pathogen or its antigens persist in the dental biofilm, the lesion is not contained.

On reviewing studies that included biopsies, we found that IFN-*γ* was associated with generalized aggressive periodontitis (PDagG) [50] as well as with advanced stages and periodontal pockets up to 6 mm [43, 45, 57]. Recently, Yue et al. [49] identified a positive correlation between the presence of IFN-*γ* in saliva and gingival crevicular fluid and the clinical parameters of individuals with PDag. According to Lappin et al. [60], this mediator is involved in the progression of PD, with a decrease in its gingival crevicular fluid levels after nonsurgical therapy [69].

Although the literature review revealed distinct methodologies and values for clinical measures that characterize PDs (e.g., CPD, PDag, and severe and moderate periodontitis), there are concordant results about the influence of dental treatment on the pattern of inflammatory mediators [12, 69–72].

Given the complex interrelation between inflammatory mediators and immune system cells, studies have suggested the participation of other cellular subtypes such as regulatory T cells, Th3 cells (which have a immunosuppressant profile), and Th17 cells (which have a proinflammatory profile) for better understanding periodontal infections [35, 57, 73–76] and the evolution of the infection in leprosy reactional states [10, 62, 67, 77–79].

There is evidence that Th17 cells, once stimulated in PDs, produce a variety of mediators such as IL-17 and TNF-*α*, correlated to the formation of osteoclasts, bone resorption, and loss of clinical attachment. This is associated with CPD and PDag [47, 57, 58, 65, 70, 73–75].

In reactional episodes, IL-17 seems to be involved in the development of T1R [77, 79].

### 4.3. TNF-*α* and IL-1*β*

As previously described, TNF-*α* and IL-1*β* are produced by macrophages mainly activated by lipopolysaccharides in the cell wall of gram-negative bacteria; they are among the main mediators responsible for an acute inflammatory response. These mediators participate in tissue remodeling and bone resorption in addition to stimulating angiogenesis and promoting fibroblast activation. Both mediators were identified in biopsy specimens and serum of individuals with PD during the occurrence of reactional episodes [12, 30, 39, 40, 64], whereas TNF-*α* was detected in saliva and the gingival fluid of individuals with dental infections [12, 40, 56, 80–84].

In dental infections, TNF-*α* was associated with periapical granulomas [85], radicular cysts [86], acute periapical lesions [87, 88], and, mainly, PDs [42, 49, 50, 56, 70, 89–93].

On the one hand, high serum TNF-*α* levels were shown to be associated with the extent and severity of disease [82, 94] and with the clinical parameters of individuals with PDagG and CPD [42, 49, 70, 90, 91, 95–99]; on the other hand, Tokoro et al. [89] reported high serum TNF-*α* and IL-1*β* levels in gingivitis. Even after dental therapy, the TNF-*α* levels remained high compared to the control group.

On studying the immune pattern in cases of leprosy infection, Foss [61] found an increase in TNF-*α* production associated with high levels of C-reactive protein, suggesting that TNF-*α* was involved in the inflammatory reaction of erythema nodosum.

Elevated levels of TNF-*α*, IL-6, and IL-1*β* in serum and in the lesions of patients with T2R seem to be associated with the clinical manifestations of T2R [9, 100]. Motta et al. [12] suggested that the systemic inflammatory effects triggered by IL-1*β* in individuals with leprosy and dental infection can also contribute to the triggering of erythema nodosum leprosum. In this review, we found that TNF-*α* was associated with both T1Rs and T2Rs [30, 39, 40, 64].

The dynamic interaction between the cells of innate and adaptive immunity, the balance between Th1 and Th2 lymphocyte subpopulations, and the presence of molecular mediators and their receptors seem to determine the pattern of the immunological response of PDs and leprosy reactions, controlling or amplifying the inflammatory processes [26, 36, 70, 101].

Although most studies considered in this systematic review have used the classification defined by the American Academy of Periodontology, including gingivitis, CPD, and PDag (localized or generalized), there were differences in the definition of periodontitis, the criteria to establish the depth of the periodontal pocket and the loss of clinical attachment, and the age group of the participants. For example, 28% of the articles described PD as soft, moderate, or severe. Further, some studies had a small sample size for both PDs and leprosy reactions.

According to Buduneli and Kinane [102], the depth of the periodontal pocket and gingival bleeding after probing are the most reliable parameters, not only for diagnosis, as they are key indicators of periodontal tissue destruction, but also for disease prognostication. Some authors suggest that the heterogeneity in the definitions of periodontitis hinders the comparison of results between studies [103, 104]. Based on the literature analyzed, the need for longitudinal studies with better standardization of the population presented and a greater uniformity between techniques and experiments becomes evident. The development of molecular and chemical biomarkers with predictive and prognostic value can help in the early identification of patients at an increased risk of periodontal or leprosy diseases.

In this regard, monitoring of treatment effectiveness and the development of new instruments to monitor these infections would decrease the incidence of neural injuries and disabilities in individuals with leprosy and the early loss of dental function.

In summary, regardless of the laboratory technique used and the type of sample analyzed, the identified proinflammatory mediators involved in the immune pathologic process of dental infections and leprosy reactions, particularly IL-6 and TNF-*α*, were similar in the studies reviewed. Specifically, for leprosy reactions and PDs, IFN-*γ* and IL-*β*-1 were significant. This pattern was reflected in serum, and the presence of IFN-*γ* and IL-1*β* was associated with CPD.

## Figures and Tables

**Table 1:** Frequency of articles published between January 1, 1996, and December 31, 2013, that were selected for the systematic review regarding dental infections, leprosy reactions, and the types of investigated inflammatory mediators.

Inflammatory mediators	Type of article			
	Dental infections		Leprosy reactions	
	*n* = 68^*∗*^	%	*n* = 10^*∗∗*^	%
IL-1*β*	29	42.7	3	30.0
TNF-*α*	25	36.8	7	70.0
IL-6	24	35.3	4	40.0
IFN-*γ*	17	25.0	5	50.0
IL-4	15	22.0	4	40.0
IL-10	13	19.1	4	40.0
IL-17^§^	13	19.1	3	30.0
IL-8	12	17.6	2	20.0
IL-2	11	16.2	1	10.0
IL-12	6	8.8	2	20.0
IL-1*α*	6	8.8		
IL-18	5	7.3		
IL-23	4	5.9		
IL-5	4	5.9	2	20.0
IL-11	4	5.9		
TGF-1*β*	4	5.9	1	10.0
IL-13	4	5.9	1	10.0
IL-15	3	4.4	1	10.0
IL-7	2	2.9	1	10.0
IL-12p40	2	2.9		
IL-12p70	2	2.9		
IL-23p19	2	2.9		
IL-9	1	1.5	1	11.1
IL-12p35	1	1.5		
IL-21	1	1.5		

^*∗*^Number of articles about dental infections.

^*∗∗*^Number of articles about leprosy reactions.

^§^Included in the cytokines IL-17A and IL-17F.

**Table 2:** Articles selected for the systematic review on dental infections and the presence of inflammatory mediators in serum (a), biopsy specimens (b), and gingival crevicular fluid (GCF) (c) according to the publication year, author, type of sample, and obtained results.

Year	Authors	*N*	Significant results
(a) Dental infections and Presence of mediators in serum			

2011	Kinney et al.^*∗*^ [105]	83 (PD)	IL-1*β*, MMP-8, and MMP-9 were strongly correlated with PD status.
	Özçaka et al.^*∗*^ [106]	22 (CPD), 21 (C)	Individuals with CPD had lower IL-17 levels in saliva.
	Robati et al. [51]	25 (PDagG) 25 (C)	Low levels of IL-4 were associated with PDagG, and IL-6 levels were high compared with the control group.
	Sánchez-Hernández et al.^*∗∗*^ [35]	18 (CPD), 12 (PDag), 9 (C)	Individuals with PDag had higher IL-12 levels in gingival tissue and serum. Those with CPD had higher serum IL-18 concentrations than controls.

2010	Duarte et al. [70]	14 (PDagG) 14 (CPDg); 14 (C)	After periodontal treatment, the serum TNF-*α* concentration remained high in the PDagG group
	Schenkein et al. [73]	53 (PDagL), 49 (PDagG), 67 (C)	IL-17 was associated with the loss of clinical insertion. Individuals with PDagG or PDagL had higher serum IL-17 concentrations.

2008	Abdolsamadi et al. [107]	40 (LPC) 40 (C)	Production of IL-6 in LPC could be used as a marker of chronic apical periodontitis.
	de Queiroz et al. [108]	17 (CPD), 8 (C)	Serum levels of RANTES, MIG, and eotaxin differed between healthy individuals and those with periodontitis.

2005	Bretz et al.^*∗∗∗*^ [42]	1131 (severe, moderate, or absent disease)	High levels of plasma TNF-*α* were associated with the extent of PD and number of teeth. IL-6 levels were higher in individuals with more extensive PD than in other individuals.

2003	Górska et al.^*∗∗*^ [56]	25 (CPD) 25 (C)	Serum and gingival tissue biopsy specimens of individuals with CPD had higher levels of IL-1*β*, TNF-*α*, IL-2, and IFN-*γ* than those of the control group.

2001	Murata et al. [52]	276 individuals	The severity of PD was not associated with the average serum IL-6 concentration. Further, 54% were positive for IL-6 in serum.

(b) Dental infections and presence of inflammatory mediators in biopsy specimens			

2012	Dutzan et al. [80]	10 (CPD), 8 (C)	Individuals with CPD showed increased expression of IL-21, IL-1*β*, IL-6, IL-17, and IL-23p19 and decreased expression of IL-10 and TGF-*β*1.

2011	Dutzan et al.^†^ [109]	15 (CPD), 19 (C)	Individuals with CPD had higher IL-21 levels in gingival tissue and GCF than controls.
	Santos [57]	36 (DGC), 31 (CPD), 15 (C)	IFN-*γ* was present in the gingival tissue of all samples and was present at higher concentrations in more advanced stages.

2009	Dutzan et al.^†^ [58]	106 (moderate or advanced CPD), 25 active sites; 25 inactive sites	The IFN-*γ* level in gingival fluid was higher than at the active site. Progressive periodontal lesions in individuals with CPD had higher expression of IFN-*γ* and had more frequent IFN-*γ* expression.
	Fukada et al. [110]	20 (GP), 10 (cysts), 8 (C)	Granulomatous tissue showed increased expression of IL-10, whereas periapical tissue with granuloma and cyst had similar expressions of IFN-*γ* and IL-4.
	Ohyama et al. [74]	15 (PD) 11 (C)	Individuals with PD had higher levels of IL-23 and IL-12 in periodontal lesions than the control group.

2008	Honda et al. [75]	24 (PD) 23 (G)	Expression of IL-17A mRNA was higher than that of IL-17F mRNA. The expression of IL-17A differed in gingivitis and periodontitis.
	Menezes et al. [85]	57 (GP) 38 (C)	Periapical granulomas showed higher TNF-*α*, IL-10, and RANKL mRNA expression than healthy periodontal tissues.

2007	Johnson and Serio [43]	59 (BP = 3 mm and SG) 73 (BP = 4–6 mm) 53 (BP > 6 mm) 58 (C)	Affected gingival tissue (3–6 mm) showed higher concentrations of IFN-*γ*, IL-2, IL-4, IL-6, IL-10, and IL-13 than controls. IL-6 showed a positive correlation with sulcular impairment.
	Jurisic et al. [86]	43 (CR), 15 (keratocysts)	A higher concentration of TNF-*α* was observed in radicular cysts.
	Kokkas et al. [44]	6 (reversible pulpitis) 6 (irreversible pulpitis), 6 (C)	The increase in TNF-*α* gene expression was associated with irreversible pulpitis compared with the control group. TNF-*α* was positively associated with the severity of clinical parameters.
	Brekalo Pršo et al. [87]	Group I: 15 (sensitive LP), Group II: 15 (insensitive LP), 15 (C)	Groups I and II had higher levels of TNF-*α*. Symptomatic periapical tissues had higher levels of IL-6 than asymptomatic periapical tissues and controls.

2006	Honda et al. [59]	25 (CPD) 23 (G)	Individuals with periodontitis had higher levels of IL-1*β*, IFN-*γ*, RANKL, HSP60, and TGF-*β*1. The levels of IL-4 were slightly higher in periodontitis than in gingivitis.

2005	Johnson and Serio [45]	36 (BP = 3 mm and SG) 39 (BP 4–6 mm) 15 (BP > 6 mm) 42 (C)	Concentrations of IL-2, IL-4, IL-6, IL-10, IL-18, and IFN-*γ* were higher in biopsy specimens from tissue adjacent to BP of 4–6 mm than in controls. Higher concentrations of IL-6 and IL-18 were noted adjacent to sites with a probing depth >6 mm than in healthy sites.
	Rodríguez and López [46]	13 (G), 9 (CPD) 13 (C)	Individuals with gingivitis and periodontitis had higher concentrations of IL-6 in gingival tissues than in healthy tissues.

2004	Johnson et al. [47]	19 (BP = 3 mm and SG) 24 (BP 4-5 mm) 11 (BP ≥ 6 mm) 31 (C)	IL-6 concentration increased with probing depth; the IL-11 concentration was higher around BP = 3 mm, and the IL-17 concentration was higher around BP of 4-5 mm compared with the other sites.

2003	Zehnder et al. [111]	11 (severe caries, symptomatic), 13 (C)	Teeth with severe caries showed a higher expression of IL-6, IL-8, and IL-18.

2002	Pezelj-Ribaric et al. [112]	20 (irreversible pulpitis) 20 (extensive caries restoration), 20 (C)	Teeth with irreversible pulpitis showed higher concentrations of TNF-*α* than controls.

2001	Lappin et al. [60]	10 (PIP) 10 (CPD)	IFN-*γ* and IL-2 were involved in disease progression, suggesting a modulator role in the inflammatory response.

2000	Danin et al. [113]	25 (LPC)	TGF-*β* per mg tissue was correlated with the diameter of the lesions.

1999	Barkhordar et al. [114]	6 (pulpitis), 6 (LP) 8 (C)	Samples of the periapical and inflamed pulp tissue showed medium levels of IL-6, which were higher compared with control levels.
	Huang et al. [115]	Teeth (irreversible pulpitis and C)	Teeth with irreversible pulpitis had higher levels of IL-8 than those with healthy pulp.

1998	McGee et al. [48]	*N* = 8 *n*_I_: BP ≤3 mm *n*_II_: BP with 4–6 mm; *n*_III_: BP >6 mm	There was a higher concentration of IL-8 around BP ≤3 mm and a higher concentration of IL-6 and IL-1*β* around BP >6 mm.
	Shimauchi et al. [81]	29 teeth with pulp exudates (EP) (symptomatic and asymptomatic)	There was a positive correlation between IL-1ra and IL-1*β*, at relatively higher levels of IL-1ra when compared with IL-1*β*.

1997	Rauschenberger [116]	12 (irreversible pulpitis), 17 (C)	IL-2 concentrations differed significantly between inflamed pulp tissue and healthy pulp tissue.
	Roberts et al. [117]	17 (CPD)	TNF-*α* and IL-1ra mRNA expression were higher in CPD than in healthy gingival tissue.
	Roberts et al. [118]	34 (CPD) 5 (C)	TNF-*α* mRNA expression was higher in CPD than in controls. IL-1*β*, IL-1ra, and IL-1*α* were seen more often in healthy tissue.
	Tokoro et al. [89]	13 (moderate or advanced (PD), 5 (G)	Gingival tissue with periodontitis showed a predominant expression of IL-4 and IL-5. There was a predominance of IL-1*α*, IL-1*β*, and TNF-*α* in gingivitis.

(c) Dental infections and presence of inflammatory mediators in GCF and saliva			

2013	Ertugrul et al. [90]	21 (PDagG), 21 (CPD) 21 (G), 21 (C)	PDagG had higher total levels of IL-8 in GCF than CPD, G, and controls. Levels of IL-1*β* and TNF-*α* were higher for the group with PDagG.
	Rathnayake et al. [82]	441 (PD)	IL-1*β* can be used as a marker in PD. Individuals with severe periodontitis showed a higher concentration of IL-1*β*.
	Yue et al. [49]	40 (PDag) 40 (C)	In PDag, there were higher concentrations of IL-1*β*, IL-2, IL-6, IFN-*γ*, and TNF-*α* in saliva and GCF.

2012	Ay et al. [119]	20 (PDagG), 18 (C)	The frequency of IL-11 was lower in the group with PDagG, and the concentration of IL-17 was lower than in the control group.

2011	Chaudhari et al. [95]	30 (CPD) 30 (C)	IL-1*β* was positively correlated with the following clinical parameters: bleeding on probing, pocket depth, periodontal disease rating, and tooth mobility.
	Garrido Flores et al. [88]	14 (PAA), 14 (C)	Higher TNF*α* concentrations were noted in gingival sites of teeth with PAA than in the control group.
	Kaushik et al. [83]	28 (CPDg), (C)	Individuals with PD had a medium level of elevated IL-1*β* compared with the control group.
	Shaddox et al. [50]	34 (PDagL) 9 (C)	Patients with PDag had higher levels of TNF*α*, IFN*γ*, IL1*β*, IL2, IL6, IL10, and IL12p40 than healthy individuals.
	Stashenko et al.^††^ [120]	103 (PIP_1_), 42 (PIP_2_) 45 (C)	Levels of IL-1*β* in GCF increased according to the severity of PD.

2010	Burgener et al. [121]	40 teeth (PA) 40 teeth (C)	Teeth with apical lesions had higher levels of IL-1*β* in the gingival fluid than the controls.
	Fitzsimmons et al. [122]	430 (moderate or severe PD), 509 (C)	PD was independently associated with higher levels of IL-1*β* and C-reactive protein.
	Perozini et al. [96]	12 (CPD), 12 (G) 12 (C)	IL-1*β* concentrations were higher in CPD than in the other groups. IL-1*β* levels were positively correlated with PD, the volume of gingival fluid, and pocket depth.
	Teles et al. [97]	20 (CPD) 20 (C)	Clinical parameters (PD, BOP, vol GCF, R, and Sup) were positively correlated with the levels of IL-1*β* and IL-8 in GCF.
	Teles et al. [98]	31 (PDagG) 25 (C)	PDagG had higher average levels of IL-1*β*. There was a tendency for levels of IL-2 and IL-13 to be higher in PDagG.

2009	Ay et al. [123]	40 (CPD): BP ≤4 mm; BP ≥5 mm 20 (C)	The total rate and concentration of IL-11 and IL-17 were lower in the group with BP ≥5 mm.
	Bastos et al. [91]	14 (PDag) 13 (C)	TNF-*α* concentrations were higher in PDag than in controls.
	Fitzsimmons et al. [124]	511 (moderate or advanced PD), 562 (C)	There were higher levels of IL-1*β* and PCR in individuals with PD. Clinical parameters were positively correlated with biomarker levels.
	Pradeep et al. [71]	20 (CPD), 20 (G), 20 (C), 3 individuals after treatment	IL-18 levels increased according to the severity of periodontal disease, decreasing after the treatment.
	Teles et al. [125]	74 (CPD) 44 (C)	Mean salivary levels of IL-8 were positively correlated with probing depth and the average percentage of sites with bleeding on probing.

2008	Frodge et al. [92]	35 (PD) 39 (C)	Individuals with PD had higher levels of TNF-*α*.
	Tóbon-Arroyave et al. [126]	30 (CPD) 18 (PDag) 18 (C)	The salivary level of IL-1*β* did not differ between groups with periodontitis, but it was higher than in the control group.
	Toker et al. [72]	15 (PDagG), 15 (C)	There were higher levels of IL-1*β* in sites with moderate or deep initial pocket than in the shallow pockets.
	Yücel et al. [84]	12 (CPD), 14 (G) 14 (C)	IL-1*β* and IL-12 concentrations in GCF were higher in CPD than in the control group.

2007	Tsai et al. [69]	17 (CPD)	Nonsurgical periodontal treatment resulted in a decrease in IFN-*γ* and an increase in IL-4.

2006	Gürkan et al. [127]	30 (PDagG), 32 (CPD), 15 (G) 16 (C)	The rate of TGF-*β*1 expression was higher in groups of individuals with PDagG and CPD than in the control group.

2003	Nicolau et al. [99]	20 (CPD) 20 (C)	Individuals with CPD showed higher concentrations of IL-1*β* in the gingival fluid compared with the controls.

2000	Guo et al. [128]	Chronic pulpitis Acute pulpitis Control	There were higher IL-8 concentrations in teeth with acute pulpitis than in those with chronic pulpitis.

1997	Ishihara et al. [94]	7 (PD) 2 (C)	IL-1*β* and IL-1*α* were associated with the severity of periodontal disease.

1996	Mathur et al. [93]	20 (PD) 20 (C)	The average rate and concentration of IL-1*α* in GCF were higher in individuals with PD than in controls. The site status is the major determinant of the cytokine levels in unhealthy sites.

*Note  1.* ^**∗**^Analysis in serum and saliva; ^**∗∗**^analysis in serum and biopsy specimens; ^*∗∗∗*^analysis in plasma; ^†^analysis in GCF and biopsy specimens; ^††^analysis in GCF and serum. PD, periodontal disease; MMP, matrix metalloproteinases; GCF, gingival crevicular fluid; *Note  2.* CPD, chronic periodontitis; C, control; PDagG, generalized aggressive periodontitis; PDag, aggressive periodontitis; CPDg, generalized chronic periodontitis; PDagL, localized aggressive periodontitis; LPC, chronic apical periodontitis; RANTES, regulated on activation, normal T cell expressed and secreted; MIG, monokine induced by gamma interferon; GP, periapical granuloma; G, gingivitis; RANKL, receptor activator of nuclear factor kappa-B ligand; BP, periodontal pocket; SG, gingival bleeding; CR, radicular cyst; LP, periapical lesion; PIP, early onset periodontitis; EP, pulp exudates; BOP, bleeding on probing; R, recession; Sup, suppuration; PAA, asymptomatic apical periodontitis; PA, periapical periodontitis.

**Table 3:** Articles selected for the systematic review on leprosy reactions and presence of mediators in skin biopsy and/or serum (a) and leprosy reaction, dental infection, and presence of cytokines (b) according to the publication year, authors, type of sample, and obtained results.

Year	Authors	*N*	Results
(a) Leprosy reaction and presence of inflammatory mediators in skin biopsy specimens and/or serum			

2013	Abdallah et al. [67]	31 (L), 6 (T1R), 6 (T2R), 43 (C)	Increased production of IL-4 in multibacillary forms can be responsible for the development of erythema nodosum leprosum. IL-17 was lower in cases than in controls.

2012	Chaitanya et al. [77]	80 (T1R), 21 (T2R), 90 (L), 94 (NL)	Serum IL-17 level increased during reactional states. There was higher elevation during T1R than during T2R and nonreactional states.

2011	Lockwood et al. [29]	299 (tissue)	TNF-*α* and TGF-1*β* were detected in 78% and 94% of the samples, respectively, and were associated with T1R.
	Madan et al. [129]	51 (L), 10 (R)	Levels of IFN-*γ*, IL-1*β*, and IL-10 were higher in T2R, whereas the TNF-*α* level was higher in T1R.

2009	Stefani et al. [40]	20 (R), 19 (L)	Potential biomarkers for T1R (CXCL10 and IL-6) and T2R (IL-6, IL-7, and PDGF-BB) were identified.

2007	Belgaumkar et al. [26]	94 (L), 5 (T1R), 1 (T2R)	Levels of IFN-*γ* were higher in T1R, whereas the T2R individuals showed higher levels of IL-6 compared to the nonreactional states.
	Iyer et al. [130]	49 (R), 82 (L), 112 (NL)	IFN-*γ* showed a greater association with the reactional states, mainly for T2R.

2004	Faber et al.^*∗*^ [131]	7 (L)	It was not possible to establish a relationship between the serum profile of cytokines and T1R.

2002	Teles et al. [39]	9 (T1R), 16 (T2R)	TNF-*α* and IL-6 were detected in all individuals in a reactional state.

1998	Moubasher et al. [64]	55 (L), 35 (R)	Individuals with T1R and T2R had higher serum levels of IFN-*γ*, TNF-*α*, and IL-1*β* than those in a nonreactional state. Higher levels of IFN-*γ* and IL-6 were noted in T1R and T2R, respectively.

(b) Leprosy reaction, dental infection, and presence of inflammatory mediators in serum			

2010	Motta et al. [12]	19 (L and OI), 19 (L without OI), 10 (C: NL and OI)	It was observed that 78.8% of individuals with leprosy and OI presented erythema nodosum and 15.8% presented with a reverse reaction. Seven days after dental treatment, the serum levels of IL-1, IL-6, and IL-10 were significantly different between the groups. The IL-6 and IL-10 levels in Group C were higher than those in the group with L and OI. Clinical improvement of the reactional episode was noted after dental treatment in 68.4% (13/19) of individuals.

*Note.* ^*∗*^Nonsignificant result; L, leprosy; T1R, type 1 reaction; T2R, type 2 reaction; C, control; NL, nonleprosy; R, reaction; OI, oral infection; PDGF-BB, platelet-derived growth factor two B (-BB) chain.
